# Supplementary material for: Doxycycline Ameliorates the Severity of Experimental Proliferative Vitreoretinopathy in Mice
Source: Int J Mol Sci. 2021 Oct 28;22(21):11670. doi: 10.3390/ijms222111670 (PMC8584209; doi:10.3390/ijms222111670)
Supplement: Supplementary file 1 [file ijms-22-11670-s001.zip › Doxycycline ameliorates PVR supplementary figures.pdf]

Doxycycline ameliorates the severity of experimental proliferative vitreoretinopathy in mice

Shun-Hua Chen[1], Yu-Jheng Lin[1], Li-Chiu Wang[2], Hsien-Yang Tsai[3], Chang-Hao Yang[4], Yu-Ti Teng[5], Sheng-Min Hsu[5,*]

1 Department of Microbiology and Immunology, College of Medicine, National Cheng Kung University, Tainan 701, Taiwan; shunhua@mail.ncku.edu.tw (S.-H.C.); ca130705@gmail.com (Y.-J.L.)
2 School of Medicine, I-Shou University, Kaohsiung 824, Taiwan; statolish@isu.edu.tw
3 Department of Ophthalmology, Tzu Chi Hospital, Taichung 427, Taiwan; tc1512901@tzuchi.com.tw
4 Department of Ophthalmology, National Taiwan University Hospital, College of Medicine, National Taiwan University, Taipei 100, Taiwan; chyangoph@ntu.edu.tw
5 Department of Ophthalmology, National Cheng Kung University Hospital, College of Medicine, National Cheng Kung University, Tainan 704, Taiwan; andyteng1026@gmail.com (Y.-T.T.)
* Correspondence: shengmin@ncku.edu.tw

Supplementary Figures

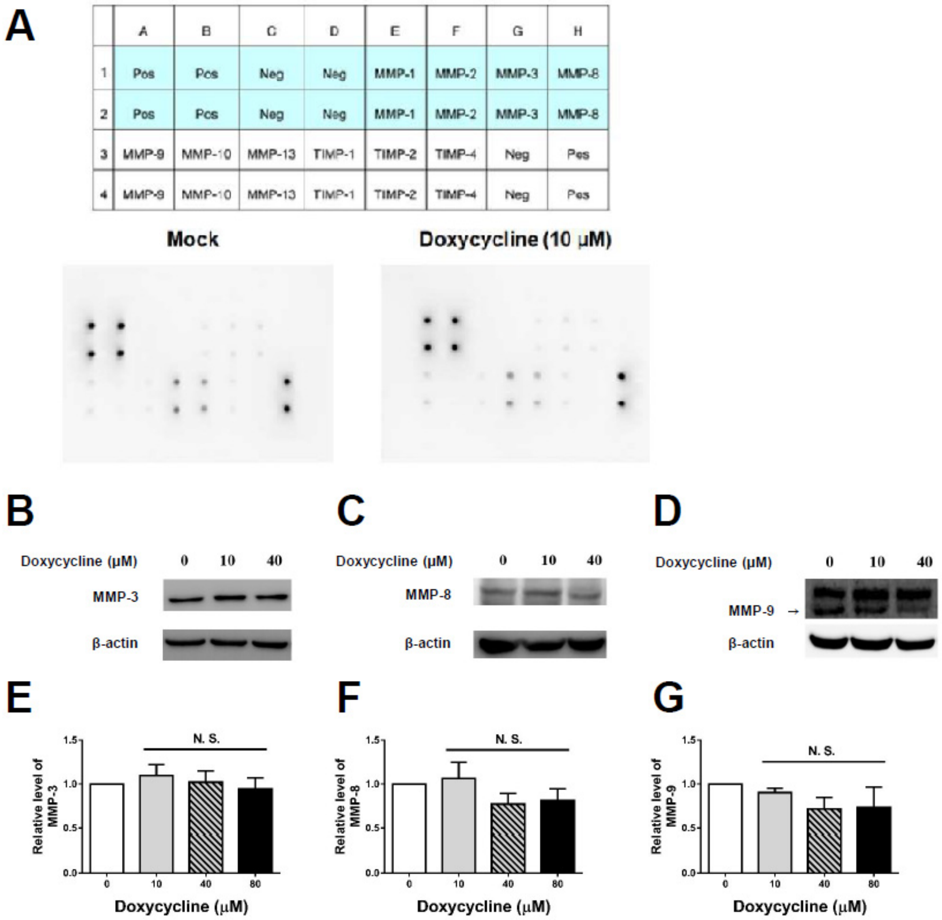

	A	B	C	D	E	F	G	H
1	Pos	Pos	Neg	Neg	MMP-1	MMP-2	MMP-3	MMP-8
2	Pos	Pos	Neg	Neg	MMP-1	MMP-2	MMP-3	MMP-8
3	MMP-9	MMP-10	MMP-13	TIMP-1	TIMP-2	TIMP-4	Neg	Pos
4	MMP-9	MMP-10	MMP-13	TIMP-1	TIMP-2	TIMP-4	Neg	Pos

Figure S1. Doxycycline fails to reduce the MMP and TIMP levels of ARPE-19 cells. ARPE-19 cells were treated with or without the indicated concentrations of doxycycline for (A) 24 or (B-D) 48 hours and assayed for MMP and TIMP levels by a human antibody array (A) and by western blotting (B-D). (B-D) The representative blots and (E-G) quantitated results are shown. (E-G) In each sample, the value of MMP was normalized with β-actin and expressed as the relative level, and the values of cells without drug treatment are set as 1. The data represent means + SEM (error bars) of 3 samples per group. N.S.: not significant.

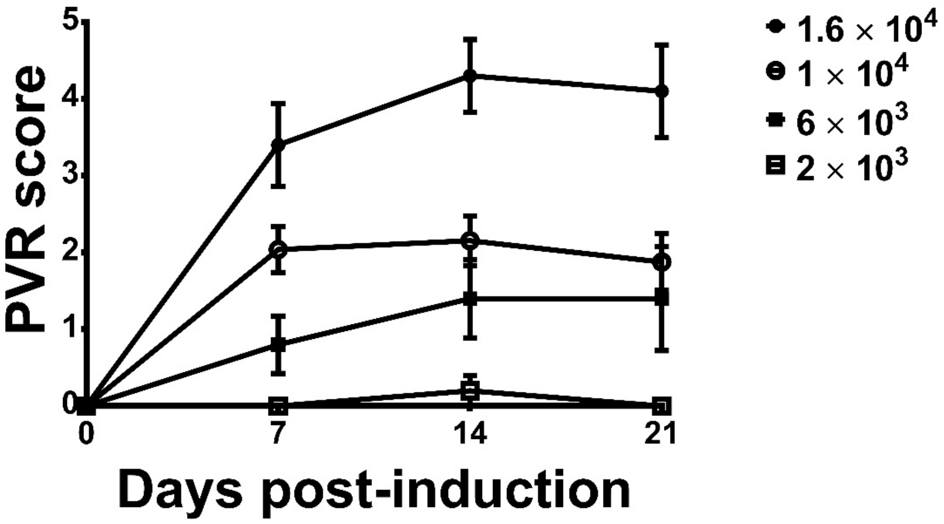

Figure S2. The dose response of ARPE-19 cells to induce PVR in mice. Mouse retinas were injected with the indicated dose of ARPE-19 cells per eye and monitored for PVR disease scores on the indicated days after induction. The data represent means ± SEM (error bars) of ≥ 4 mice per data point.

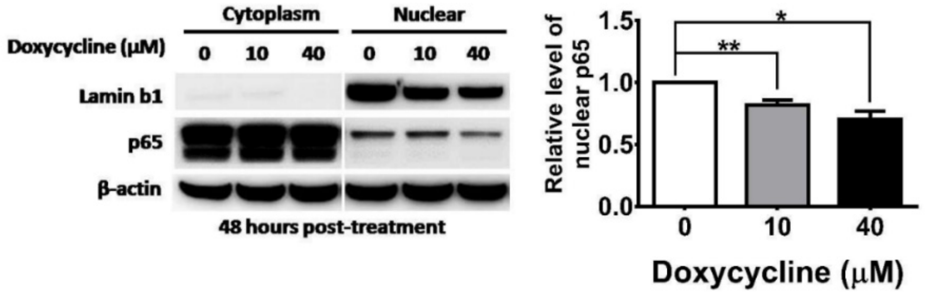

Figure S3. Doxycycline inhibits NF-κB activation of ARPE-19 cells. ARPE-19 cells were treated with or without the indicated concentrations of doxycycline for 48 hours and assayed for nuclear and cytoplasmic fractions of the NF-κB subunit, p65, β-actin, and lamin b1 (nuclear marker) by western blotting. The representative blots and quantitated results are shown. In each sample, the value of nuclear p65 was normalized to that of β-actin. The value of cells without doxycycline treatment is set as 1. The data represent means + SEM (error bars) of 3-4 samples per group. *, $P < 0.05$ and **, $P < 0.01$ via the Student's t test.

A.2.1. Assays for MMP and TIMP Levels by an Array and Western Blotting

Total proteins were extracted from ARPE-19 cells and measured for concentrations as described in *4.3*. The MMP and TIMP levels were determined by a human antibody array (Abcam) according to the instructions of manufacture. Briefly, the samples with total proteins were incubated with antibody array membranes. After a series of wash, biotin-conjugated detection antibodies and HRP-conjugated streptavidin were added to the membrane, and MMPs or TIMPs were detected by adding a detection buffer and monitored with a CCD camera. MMP levels were also detected by western blotting by the primary antibody against human MMP-3 (GeneTex, Irvine, CA, USA), human MMP-8 (Proteintech, Rosemont, IL, USA), human MMP-9 (GeneTex), and β-actin (Sigma-Aldrich) and followed by secondary antibodies (Jackson Immuno Research Laboratories).

A.2.2. Assays for NF-κB p65 in the Nucleus by Western Blotting

ARPE-19 cells were processed for the extraction of nuclear and cytoplasmic proteins by NE-PER™ Nuclear and Cytoplasmic Extraction Reagents (Thermo Fisher Scientific). Proteins in samples were measured for concentrations by the protein assay dye reagent and subjected to western blotting with the primary antibody against human NF-κB p65 (Cell Signaling Technology), lamin b1 (Abcam), or β-actin (Sigma-Aldrich) and followed by secondary antibodies (Jackson Immuno Research Laboratories).
