# Supplementary figures and images for: Doxycycline Ameliorates the Severity of Experimental Proliferative Vitreoretinopathy in Mice
Source: Int J Mol Sci. 2021 Oct 28;22(21):11670. doi: 10.3390/ijms222111670 (PMC8584209; doi:10.3390/ijms222111670)

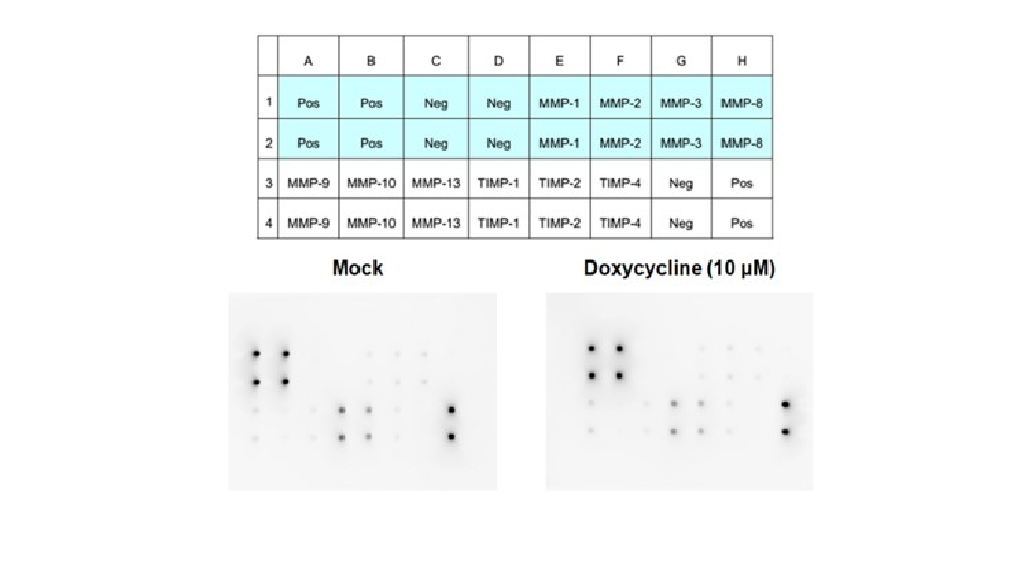

Supplement: Supplementary file 1 [file ijms-22-11670-s001.zip › SF 1A.tif]

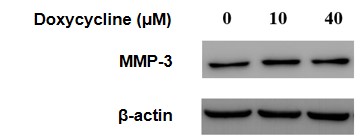

Supplement: Supplementary file 1 [file ijms-22-11670-s001.zip › SF 1B.jpg]

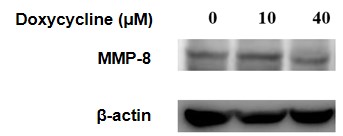

Supplement: Supplementary file 1 [file ijms-22-11670-s001.zip › SF 1C.jpg]

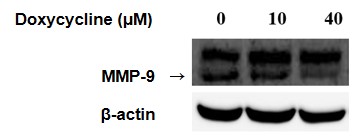

Supplement: Supplementary file 1 [file ijms-22-11670-s001.zip › SF 1D.jpg]

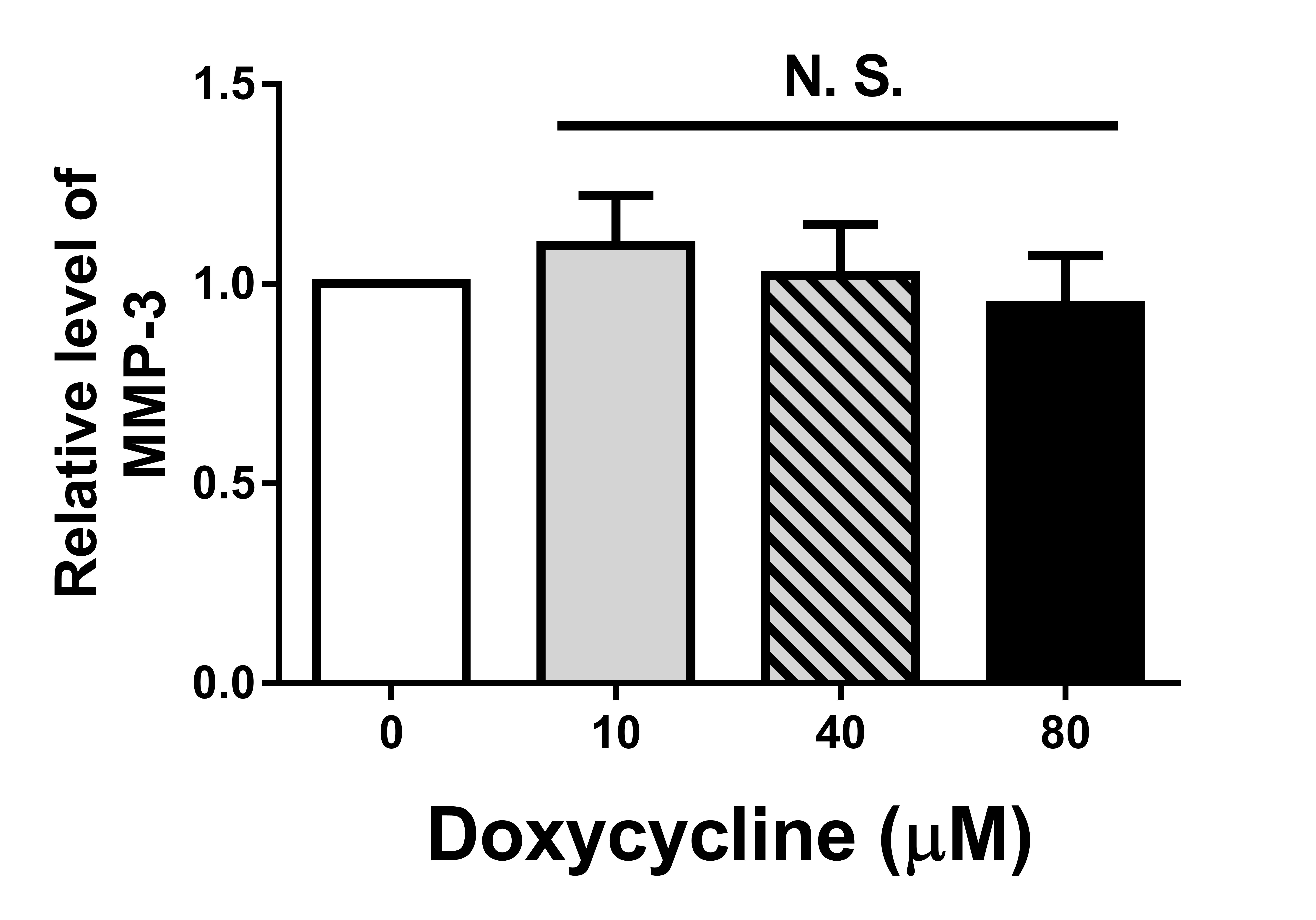

Supplement: Supplementary file 1 [file ijms-22-11670-s001.zip › SF 1E.jpg]

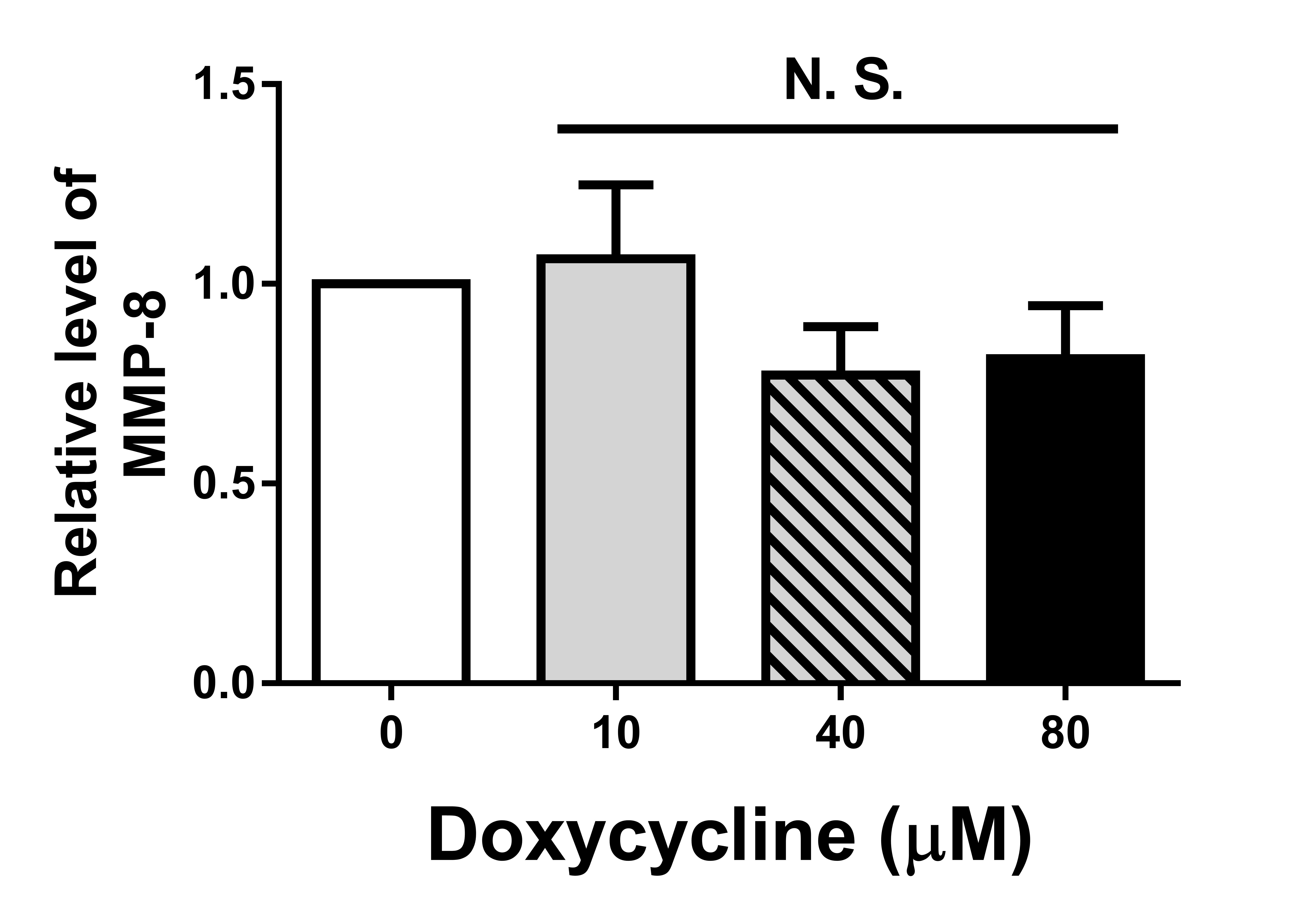

Supplement: Supplementary file 1 [file ijms-22-11670-s001.zip › SF 1F.jpg]

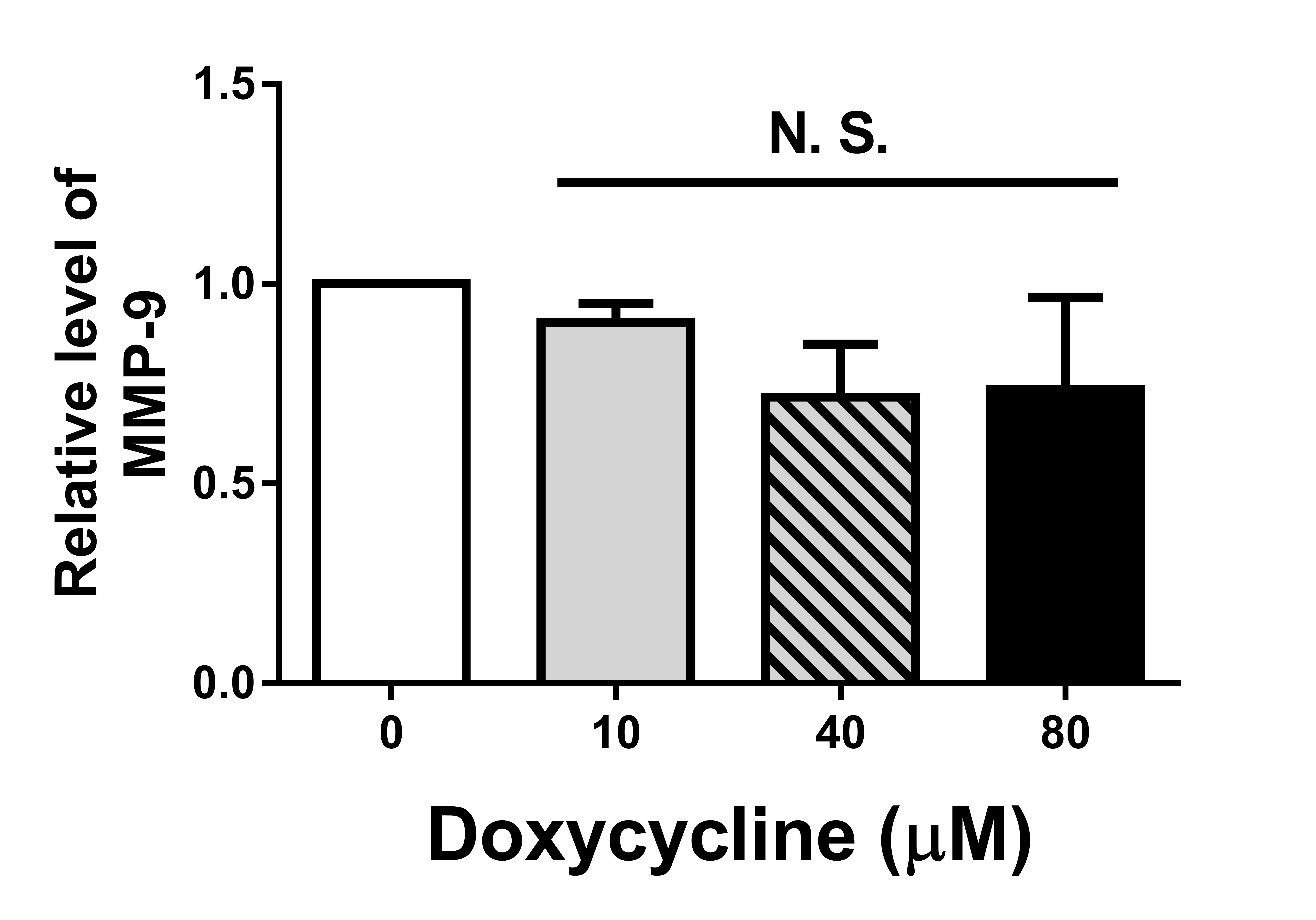

Supplement: Supplementary file 1 [file ijms-22-11670-s001.zip › SF 1G.jpg]

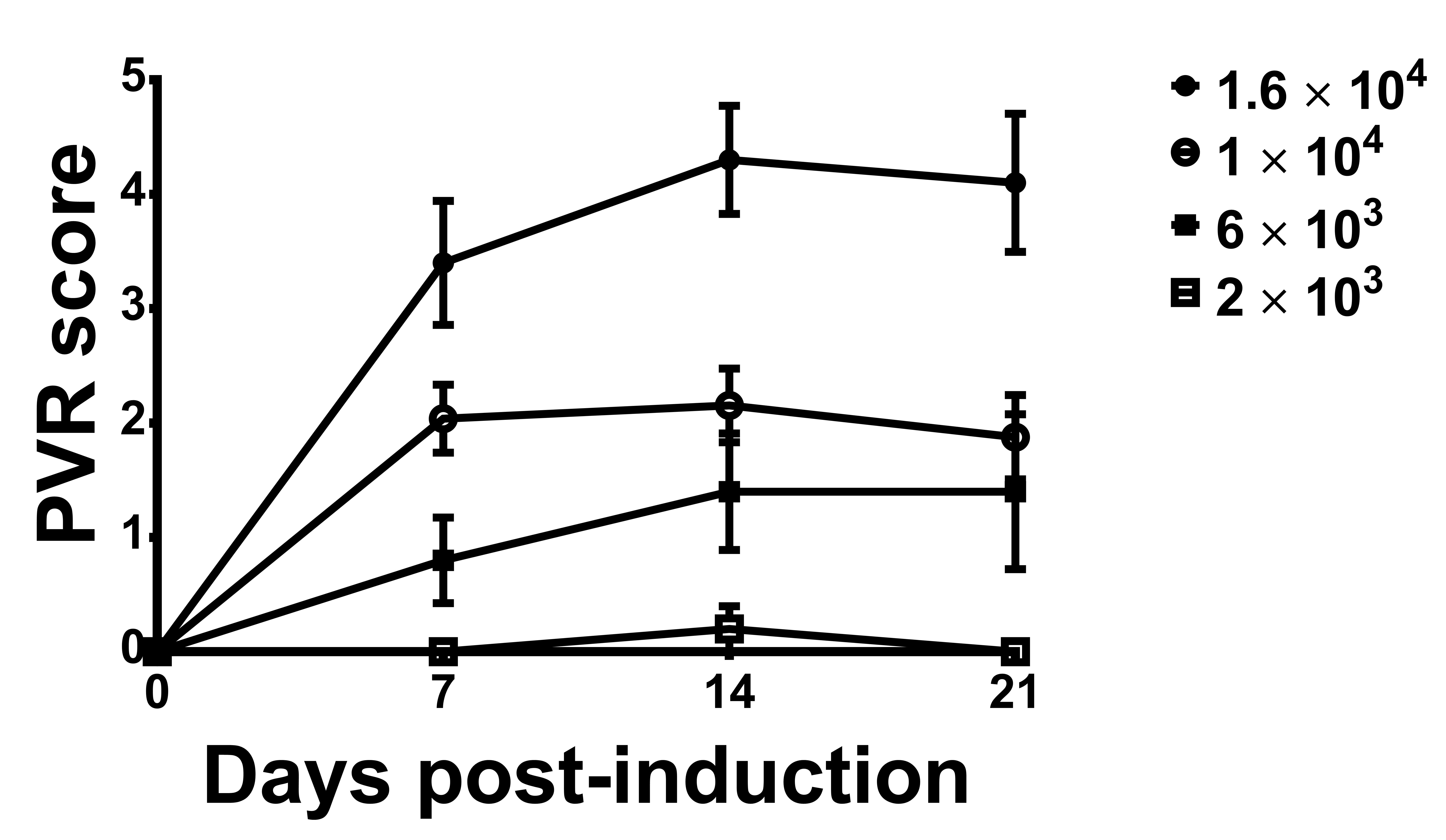

Supplement: Supplementary file 1 [file ijms-22-11670-s001.zip › SF 2.jpg]

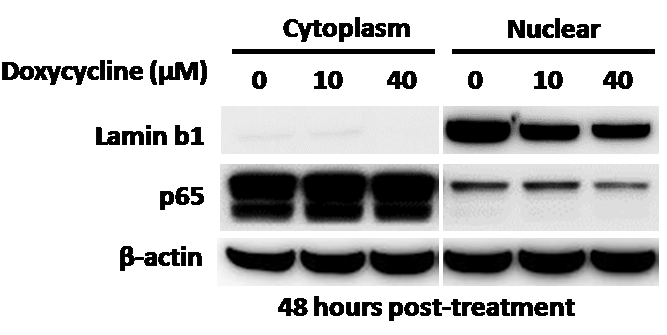

Supplement: Supplementary file 1 [file ijms-22-11670-s001.zip › SF 3 left.tif]

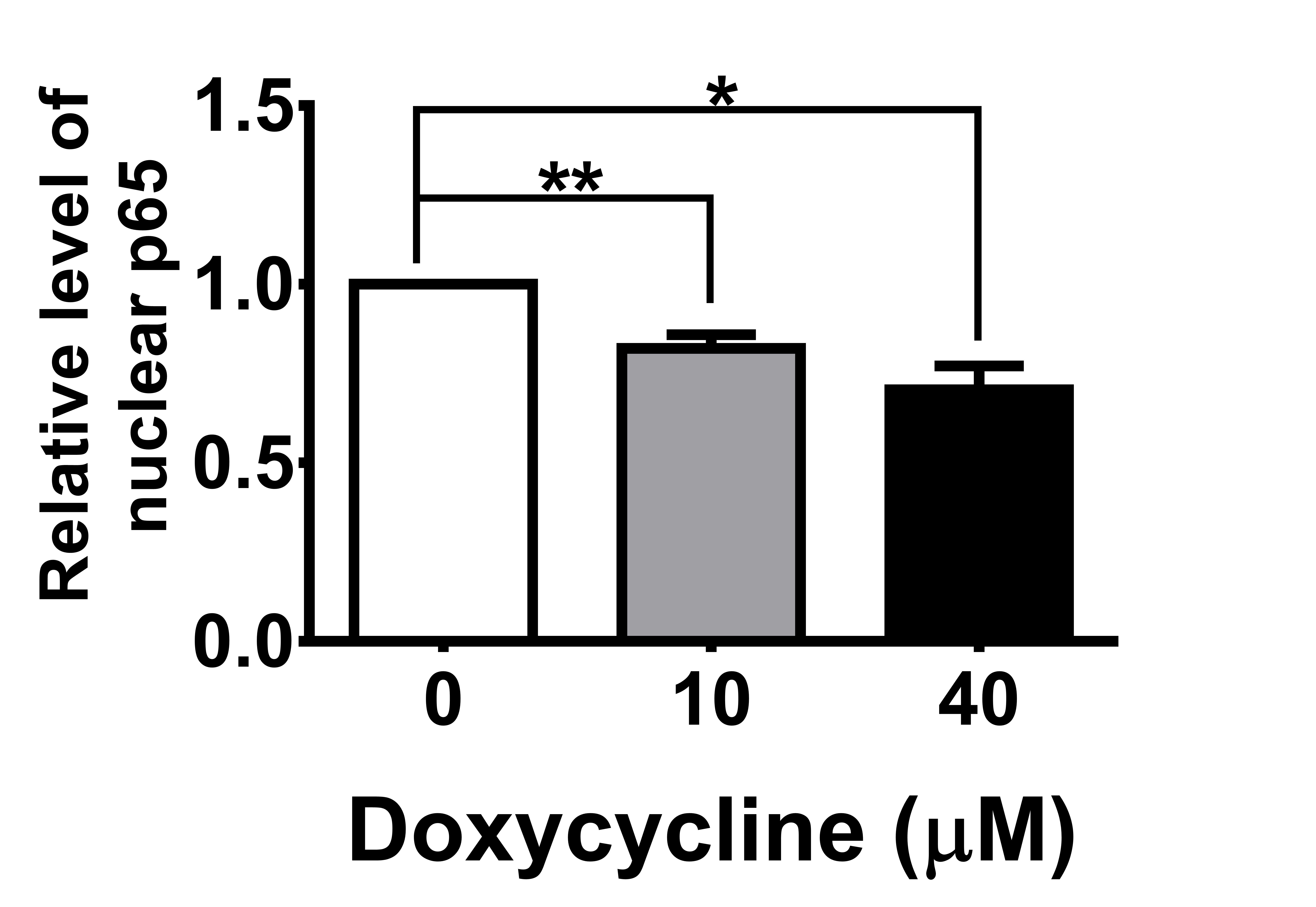

Supplement: Supplementary file 1 [file ijms-22-11670-s001.zip › SF 3 Right.jpg]
